# Supplementary material for: Tidal dynamics and mangrove carbon sequestration during the Oligo–Miocene in the South China Sea
Source: Nat Commun. 2017 Jun 15;8:15698. doi: 10.1038/ncomms15698 (PMC5481738; doi:10.1038/ncomms15698)
Supplement: Supplementary Information — Supplementary Figures, Supplementary Tables and Supplementary References [file ncomms15698-s1.pdf]

Supplementary figures

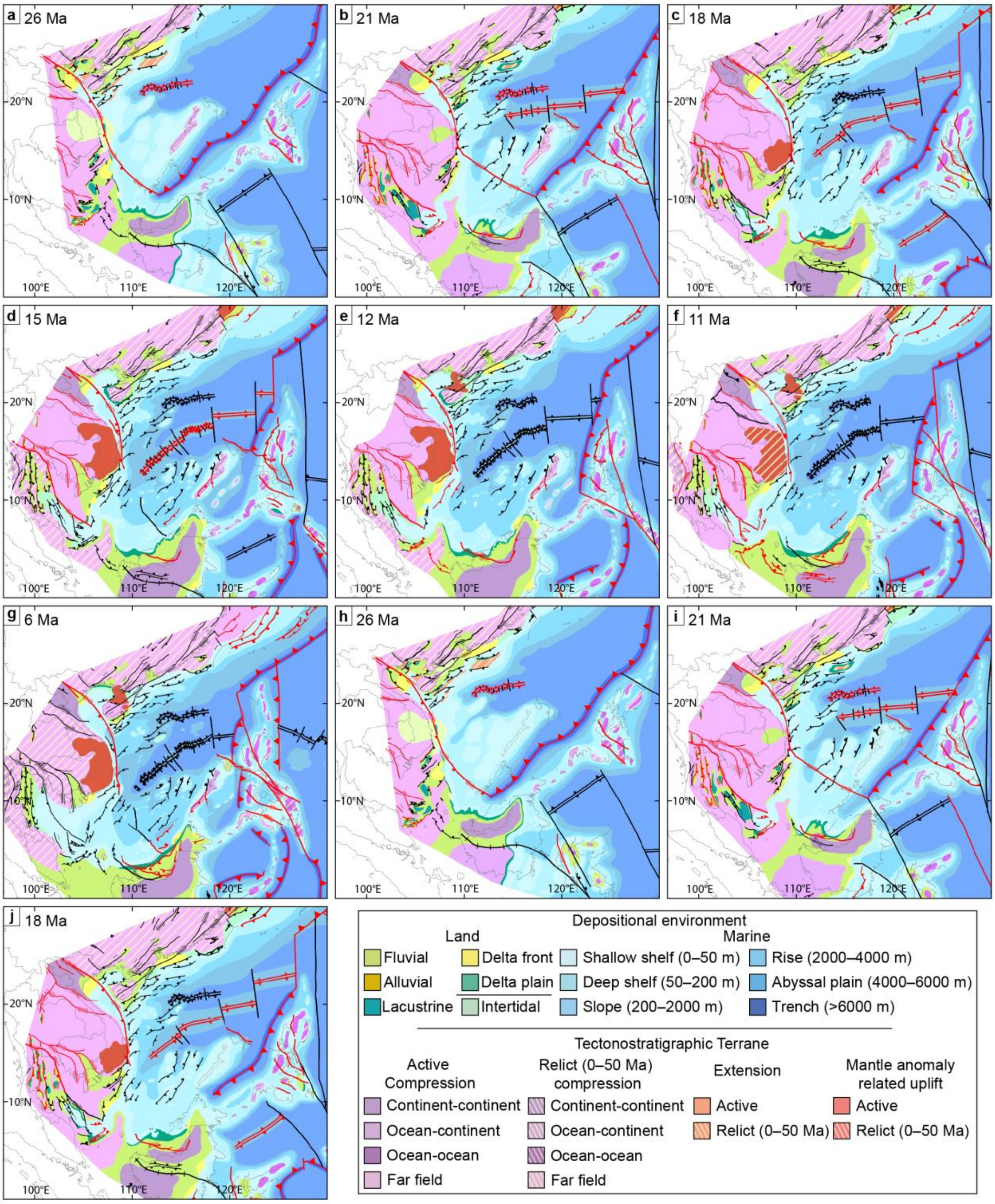

Supplementary Figure 1 | Highstand palaeogeographic reconstructions for the South China Sea (SCS) and adjacent areas from the Late Oligocene–Late Miocene. Base-case palaeogeographic interpretations were constructed for seven timeslices: **a**, 26 Ma (Chattian); **b**, 21 Ma (Aquitanian); **c**, 18 Ma (Burdigalian); **d**, 15 Ma (Langhian); **e**, 12 Ma (Serravallian); **f**, 11 Ma (Tortonian); and **g**, 6 Ma (Messinian). Alternative palaeogeographic reconstructions without Palawan were constructed for three timeslices: **h**, 26 Ma (Chattian); **i**, 21 Ma (Aquitanian); and **j**, 18 Ma (Burdigalian).

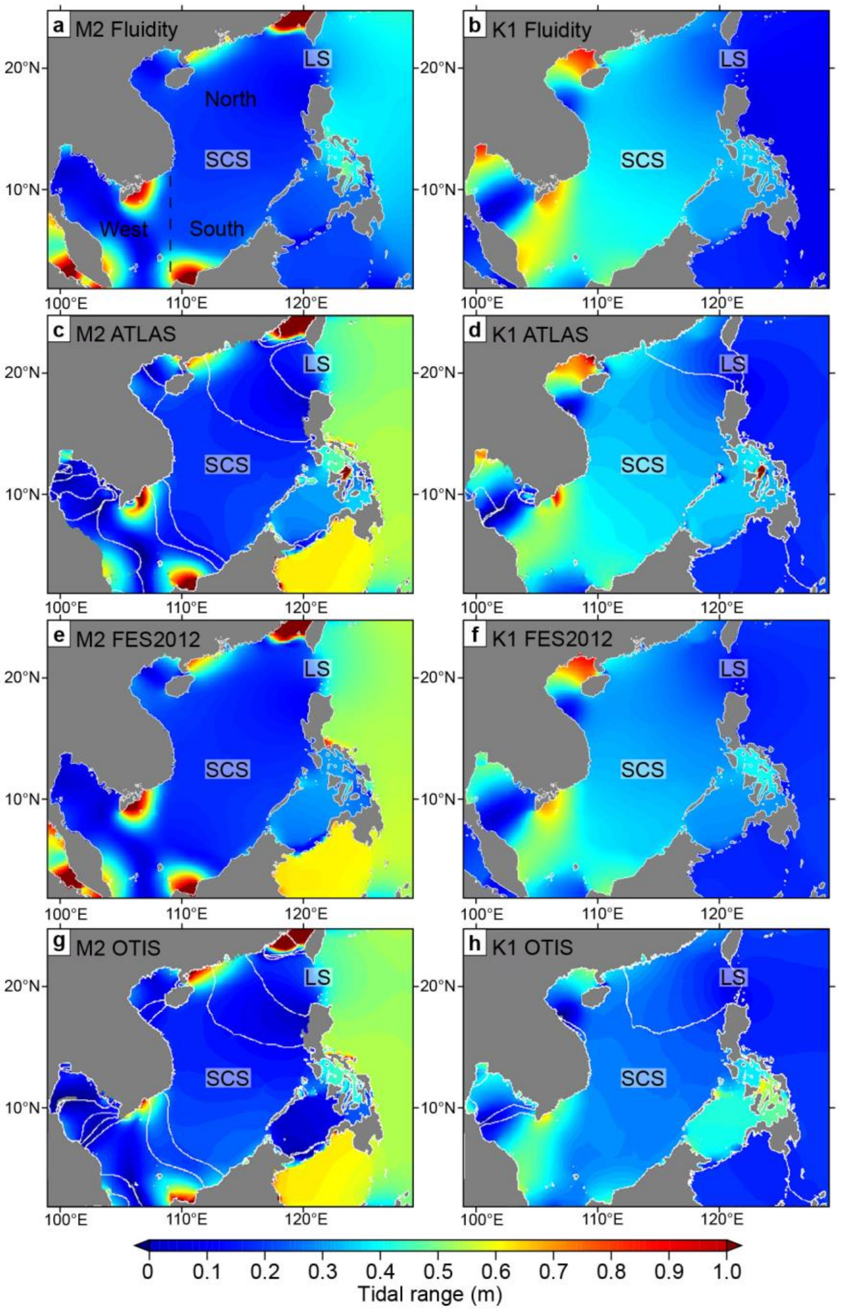

Supplementary Figure 2 | Comparison between modelled and observed M2 and K1 tide amplitudes in the modern SCS. The pattern and magnitudes of the M2 (**a**) and K1 (**b**) tides modelled using Fluidity compare favourably with the ATLAS (TPXO8) (**c**–**d**), FES2012 (**e**–**f**) and OTIS (**g**–**h**) models[1]. LS—Luzon Strait.

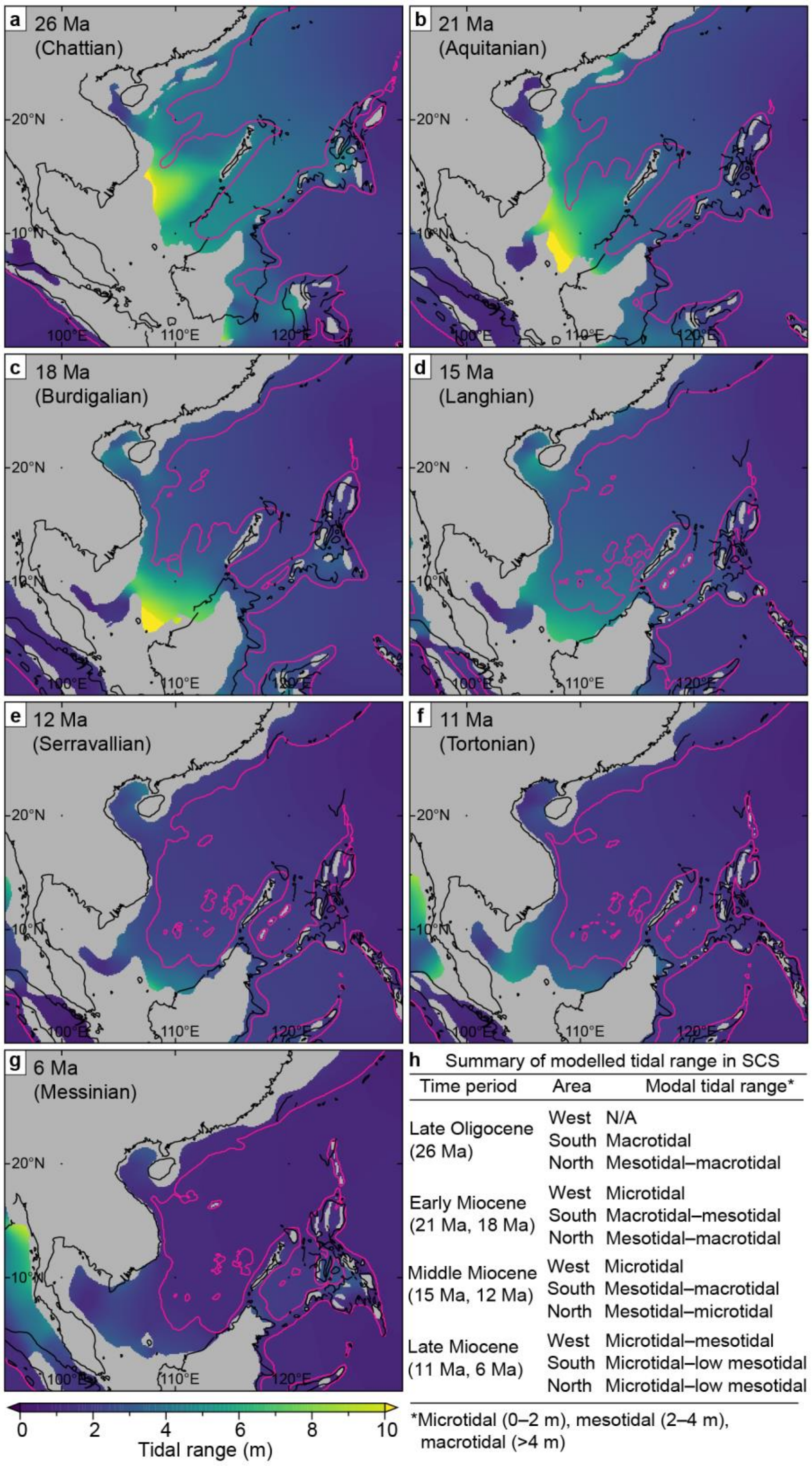

h Summary of modelled tidal range in SCS

Time period	Area	Modal tidal range*
Late Oligocene (26 Ma)	West	N/A
	South	Macrotidal
	North	Mesotidal–macrotidal
Early Miocene (21 Ma, 18 Ma)	West	Microtidal
	South	Macrotidal–mesotidal
	North	Mesotidal–macrotidal
Middle Miocene (15 Ma, 12 Ma)	West	Microtidal
	South	Mesotidal–macrotidal
	North	Mesotidal–microtidal
Late Miocene (11 Ma, 6 Ma)	West	Microtidal–mesotidal
	South	Microtidal–low mesotidal
	North	Microtidal–low mesotidal

*Microtidal (0–2 m), mesotidal (2–4 m), macrotidal (>4 m)

Supplementary Figure 3 | Model results for tidal range in the Oligocene-Miocene of the SCS area using the base-case palaeogeographic interpretation. a, 26 Ma (Chattian). **b,** 21 Ma (Aquitanian). **c,** 18 Ma (Burdigalian). **d,** 15 Ma (Langhian). **e,** 12 Ma (Serravallian). **f,** 11 Ma (Tortonian). **g,** 6 Ma (Messinian). **h,** Summary table of modelled tidal range in SCS. Refer to Supplementary Figure 2 for delineation of West, South and North areas of the SCS. The black line is the reconstructed present-day coastline. The 200 m palaeobathymetric contour (pink line) is the approximate palaeo-shelf edge.

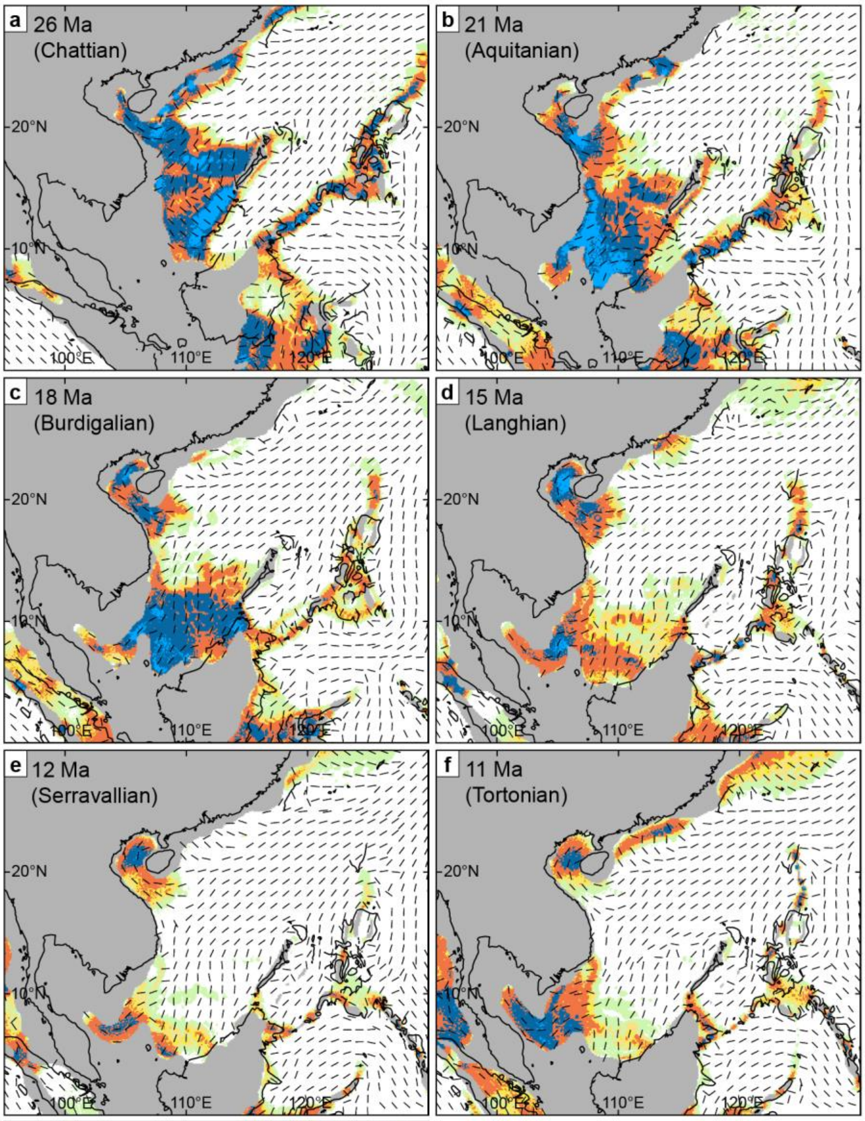

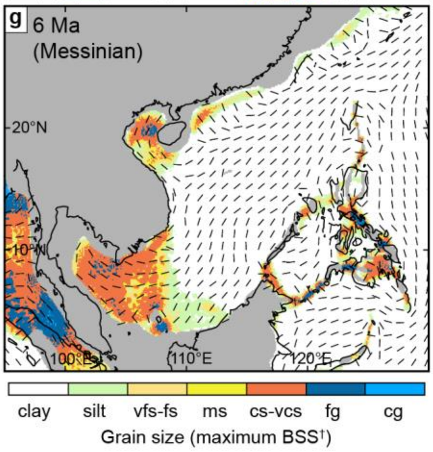

h Summary of modelled tidal BSS* in SCS

Time period	Area	Maximum BSS†
Late Oligocene (26 Ma)	West	N/A
	South	Coarse sand–coarse gravel
	North	Coarse sand–coarse gravel
Early Miocene (21 Ma, 18 Ma)	West	Medium sand–fine gravel
	South	Coarse sand–fine gravel
	North	Coarse–fine sand
Middle Miocene (15 Ma, 12 Ma)	West	Coarse sand–fine gravel
	South	Coarse–fine sand
	North	Coarse–fine sand
Late Miocene (11 Ma, 6 Ma)	West	Coarse–fine sand
	South	Silt–mud
	North	Silt–fine sand

*BSS—Bed shear stress
†Quoted as maximum modal grain size transportable by tides

Supplementary Figure 4 | Model results for maximum tidal bed shear stress, plotted as the maximum grain size that could be entrained by tides, in the Oligocene-Miocene in the SCS using the base-case palaeogeographic interpretation. a, 26 Ma (Chattian). **b,** 21 Ma (Aquitanian). **c,** 18 Ma (Burdigalian). **d,** 15 Ma (Langhian). **e,** 12 Ma (Serravallian). **f,** 11 Ma (Tortonian). **g,** 6 Ma (Messinian). **h,** Summary table of modelled maximum tidal BSS in SCS. Refer to Supplementary Figure 2 for delineation of West, South and North areas of the SCS. The thicker black line is the reconstructed present-day coastline. The thinner black lines indicate the direction of maximum tidal bed shear stress.

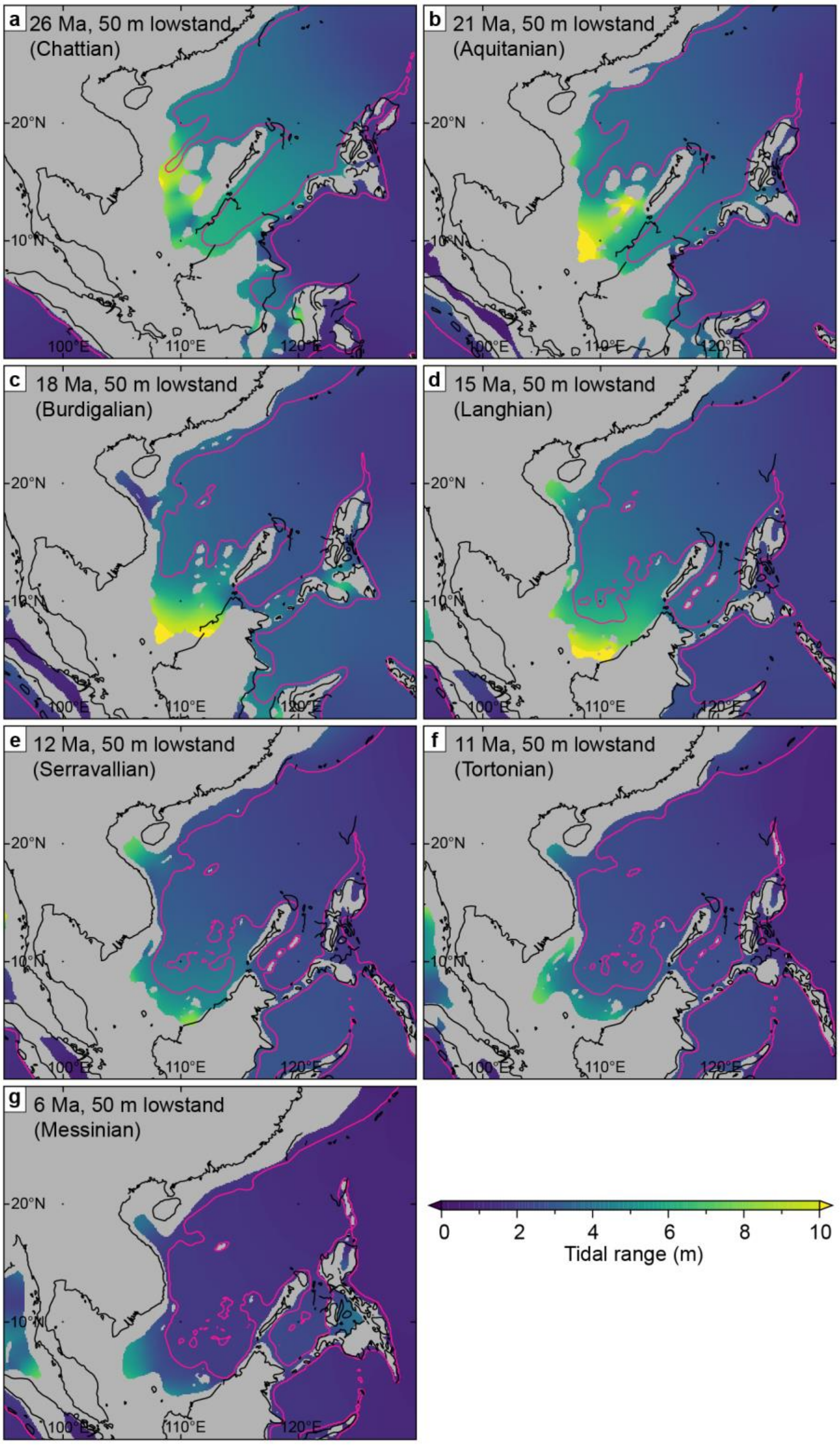

Supplementary Figure 5 | Model results for tidal range in the Oligocene-Miocene in the SCS using the base-case palaeogeographic interpretation but with mean sea level lowered by 50 m. a, 26 Ma (Chattian). **b,** 21 Ma (Aquitanian). **c,** 18 Ma (Burdigalian). **d,** 15 Ma (Langhian). **e,** 12 Ma (Serravallian). **f,** 11 Ma (Tortonian). **g,** 6 Ma (Messinian). The 200 m palaeobathymetric contour (pink line) is the approximate palaeo-shelf edge.

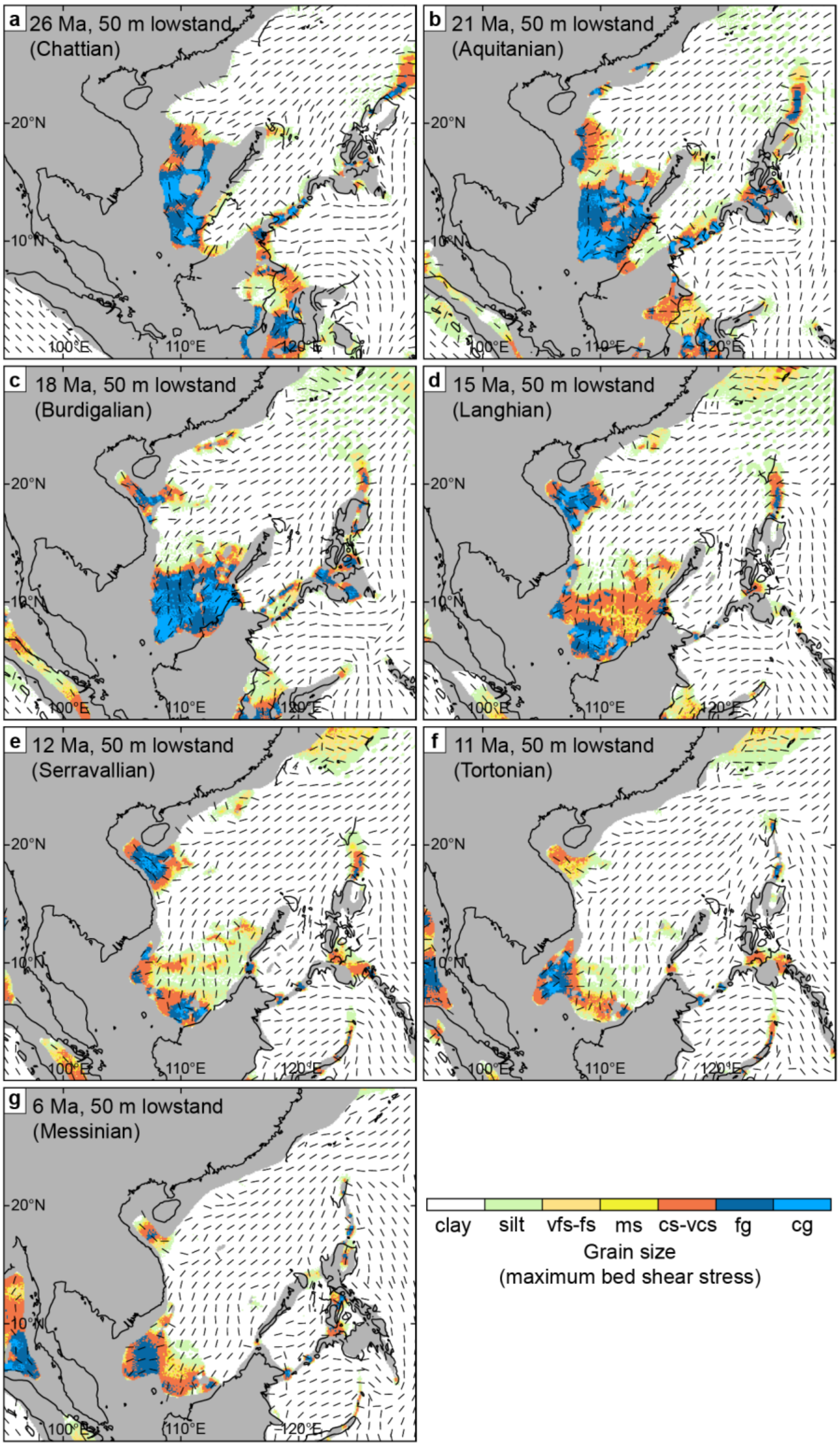

Supplementary Figure 6 | Model results for maximum tidal bed shear stress, plotted as the maximum grain size that could be entrained by tides, in the Oligocene-Miocene in the SCS using the base-case palaeogeographic interpretation, but with mean sea level lowered by 50 m. a, 26 Ma (Chattian). **b,** 21 Ma (Aquitanian). **c,** 18 Ma (Burdigalian). **d,** 15 Ma (Langhian). **e,** 12 Ma (Serravallian). **f,** 11 Ma (Tortonian). **g,** 6 Ma (Messinian). The thicker black line is the reconstructed present-day coastline. The thinner black lines indicate the direction of maximum tidal bed shear stress.

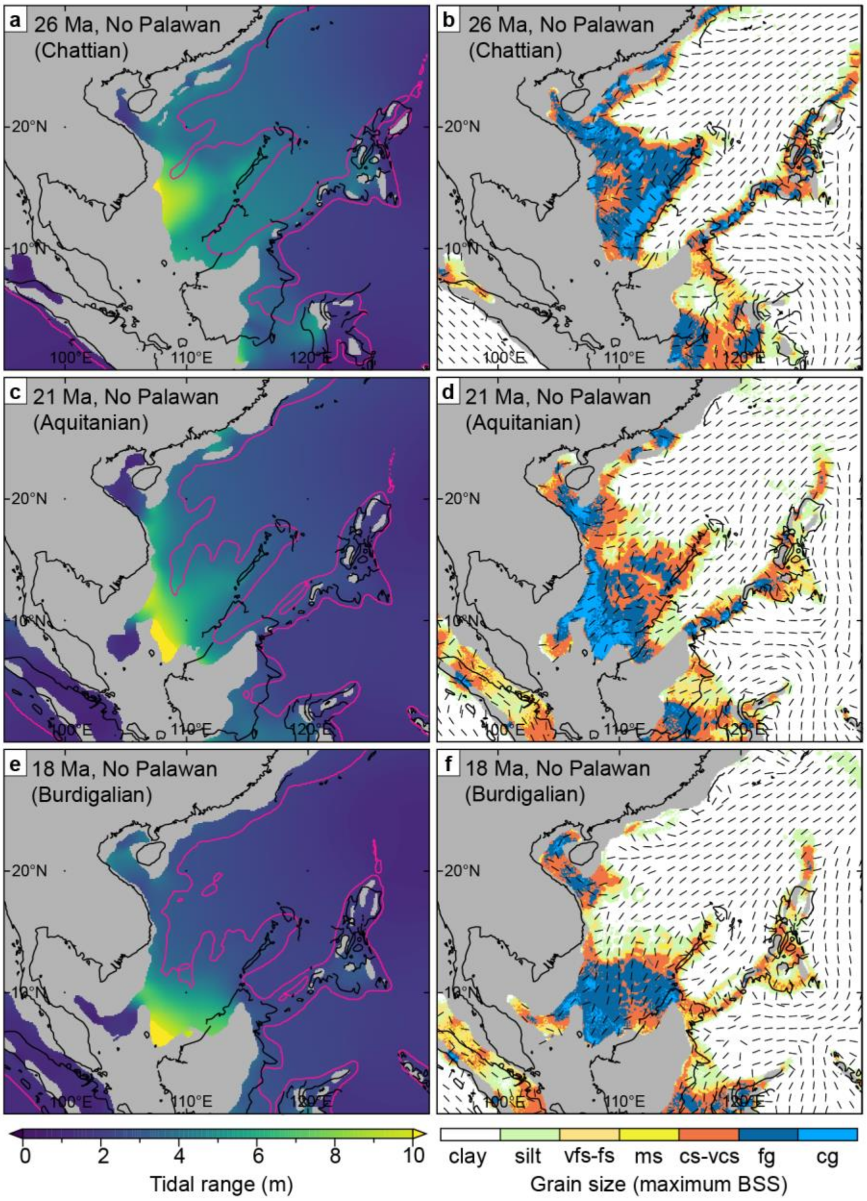

Supplementary Figure 7 | Sensitivity analysis for palaeogeographic uncertainty of Palawan in the SCS during the Late Oligocene to Early Miocene. Tidal range (**a, c, e**) and maximum bed shear stress, plotted as the maximum grain size that could be entrained by tides (**b, d, f**), are shown for 26 Ma (**a**–**b**), 21 Ma (**c**–**d**) and 18 Ma (**e**–**f**). Reconstructed palaeogeographies used in this sensitivity analysis do not include Palawan (Supplementary Figure 1h–j). The thicker black line (**a**–**f**) is the reconstructed present-day coastline. The thinner black lines (**b, d, f**) indicate the direction of maximum tidal bed shear stress. The 200 m palaeobathymetric contour (pink line; **a, c, e**) is the approximate palaeo-shelf edge.

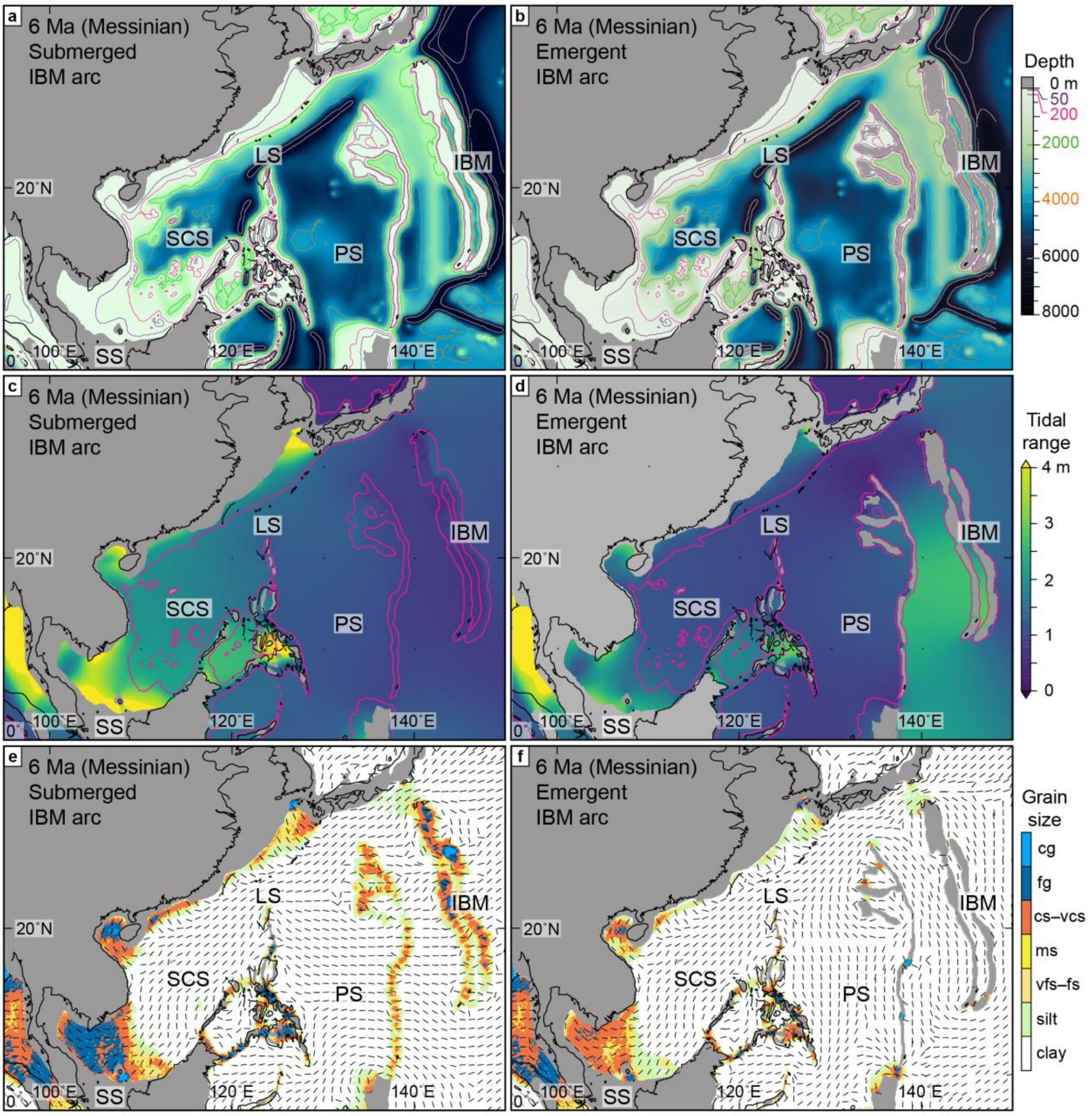

Supplementary Figure 8 | Comparison between modelled tides for a submerged and emergent (base case) Izu-Bonin-Mariana (IBM) arc in the Messinian (6 Ma). Bathymetry (**a–b**), tidal range (**c–d**) and maximum bed shear stress, plotted as the maximum grain size that could be entrained by tides (**e–f**), are shown for a palaeogeographic interpretation with a submerged (**a, c, e**) and emergent (**b, d, f**) IBM arc. The thicker black line (**a–f**) is the reconstructed present-day coastline. The thinner black lines (**e–f**) indicate the direction of maximum tidal bed shear stress. The 200 m palaeobathymetric contour (pink line; **c–d**) is the approximate palaeo-shelf edge. PS—Philippine Sea. SS—Sunda Shelf.

Supplementary tables

Supplementary Table 1 | Facies associations in studied outcrops in the Balingian and Baram Delta provinces, northwest Borneo

Facies association (FA)	Log*	Ichnofauna†	Associations	Environment and process§ interpretation	
FA 1<br>Sandier-upwards, mudstone-dominated and sharp-based, swaley cross-stratified, sandstone-dominated units	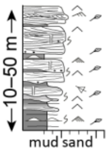	FW: BI 0–5<br>*Pl, T, Th, Ch, R, Pa*<br>S: BI 0–2<br>*Op, Pt, Pa*	Gradually or abruptly overlies FA 1–3, 5–8<br>Overlain by FA 1, 5–7 abruptly or FA 1, 8 gradually	Fully-marine, storm-dominated, open coastline delta front and flanking shoreface<br>Storm-dominated preservation, variable energy, process or sediment supply	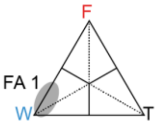
FA 2<br>Sandier-upwards, variably bioturbated heterolithic to swaley cross-stratified, sandstone-dominated units	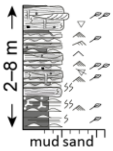	BI 1–5, highly variable abundance and diversity<br>*Op, Th, P, Pa, As, Ph, Cy, Ro, Sk*	Overlies FA 2–3, 6–9 gradually or FA 1–2 abruptly<br>Abruptly overlain by FA 2, 6–9 or gradually by FA 1–2	Mixed-energy, storm- to fluvial-dominated, tide-affected (?) delta and flanking shoreface, open or embayed coastline	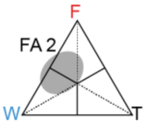
FA 3<br>Upward-coarsening, muddy to sandy heterolithic units	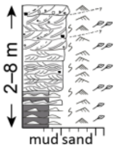	BI 1–2, locally BI 3–4 impoverished, moderate diversity<br>*Pl, T, Th, Cy, R, Pt, Sk*	Gradually or abruptly, overlies and overlain by FA 1–2, 6–9	Prograding tide-influenced/-dominated bars/deltas within a relatively wave-protected embayment or tide-dominated/ fluvial-tidal channel	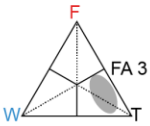
FA 4<br>Upward-coarsening, sparsely bioturbated, heterolithic to sandstone-dominated units	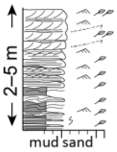	BI 0–1<br>Absent to minor, small (mm-scale) simple, low diversity (e.g. *Pl*)	Gradually overlies FA 8<br>Abruptly overlain by FA 4, 6–8	Fluvial-dominated mouth bar/delta, wave- and tide- affected	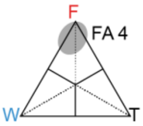
FA 5<br>Erosionally-based, fining-upwards sandstone-dominated to muddy heterolithic units	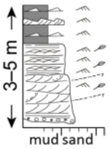	sst: absent<br>mst: BI 0–1<br>*Pl, Pa, Pt,* micro-*Op* fu	Abruptly overlies FA 4–8<br>Overlain gradually by FA 8 and abruptly by FA 1–2, 8	Fluvial-dominated channel (andabandonment), single-storey (3–5 m thick) and multi-storey (10–70 m thick)	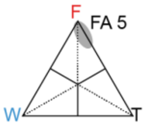
FA 6<br>Erosionally-based, fining-upwards sandstone-dominated to sandy and muddy heterolithic units	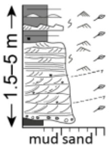	BI 0–2<br>Pl, Pa, Pt,<br>*Sk, Op,* cb	Abruptly overlies FA 6–8, 1–2<br>Overlain gradually by FA 1–3, 8 and abruptly by FA 1–2	Tide-influenced fluvial channel (and abandonment), single-storey (3–5 m thick) and multi-storey (10–70 m thick)	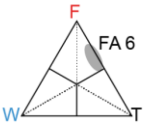
FA 7<br>Erosionally-based, fining-upwards, heterolithic-dominated units	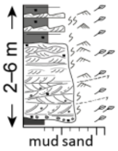	BI 0–3, locally BI 4<br>*Pl, Pa, Pt, Sk, Op,* cb	Abruptly overlies FA 8–9, FA 2<br>Overlain gradually by FA 8–9 or abruptly by FA 1–2, 8–9	Tide-dominated subtidal channel (sand- and mud-dominated)	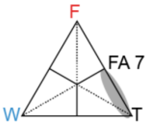
FA 8<br>Laminated to massive mudstone units with sandy interbeds	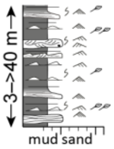	BI 0–2, locally BI 3–5<br>*Pl, T, Th, Pa*	Gradually or abruptly overlies FA 2–7, 9<br>Overlain abruptly by FA 5–7 and gradually by FA 1–4, 9	Variably tide- and storm-dominated, fluvial- influenced, marginal-marine and brackish embayment or abandoned fluvial-tidal channel	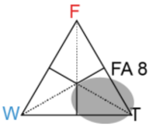
FA 9<br>Massive, carbonaceous mudstones units with minor sandy laminae and interbeds	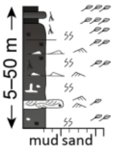	BI 4–6<br>*Th, Pl,* indistinct & rooting	Gradually or abruptly overlies FA 6–8<br>Overlain gradually by FA 8 and abruptly by FA 1–3, 6–8	Tide-dominated, mangrove-vegetated, intertidal to subtidal flat in a relatively wave-protected, marginal-marine embayment	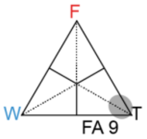

*Refer to Fig. 7 for legend. Addtional symbols are: = combined flow ripples; = climbing ripples; = dipping stratigraphic surface; • = coal fragment; • = mud clast; ◦ = lithoclast; coaly drape; = Ophiomorpha (*Op*).
†Ichnogenera recognised include *Asterosoma* (As), *Chondrites* (Ch), *Cylindrichnus* (Cy), *Ophiomorpha* (Op), *Palaeophycus* (Pa), *Phycosiphon* (Ph), *Planolites* (P), *Rosselia* (Ro), *Teichichnus* (T), and *Thalassinoides* (Th). Assemblage abbreviations: FW = Fairweather; SW = Storm. Grain-size abbreviations are: mst = mudstone; sst = sandstone. BI = Bioturbation index. mm = millimetre. Refer to ref. 2.
§Process interpretations are plotted on a Wave (W), Tide (T) and Fluvial (F) ternary plot (ref. 3).

Supplementary Table 2 | Organic carbon (OC) burial estimated in the Baram Delta Province (BDP) based on hydrocarbon volumes

		Oil			Natural Gas			Hydrocarbons	
Location	Field	Volume (bbl)	Mass of OC (Gt)	Volume of CO_2 (ppm)	Volume (tcf)	Mass of OC (Gt)	Volume of CO_2 (ppm)	Total mass of OC (Gt)	Total volume of CO_2 (ppm)
Brunei*	Seria	2.9	0.33	0.154	1.8	0.03	0.013	0.36	0.17
	Rasau	0.1	0.015	0.007	0.2	0.003	0.001	0.018	0.01
	Enggang	0.0	0.003	0.001	0.0	0.0002	0.000	0.003	0.00
	SW Ampa	2.2	0.25	0.119	14.2	0.21	0.099	0.46	0.22
	Fairley	0.5	0.061	0.029	2.5	0.037	0.017	0.098	0.05
	Egret	0.1	0.011	0.005	1.1	0.016	0.007	0.027	0.01
	Gannet	0.0	0.002	0.001	0.7	0.011	0.005	0.013	0.01
	Champion	3.0	0.34	0.159	1.8	0.026	0.012	0.36	0.17
	Peragam				0.4	0.006	0.003	0.006	0.00
	Iron Duke	0.1	0.016	0.007				0.016	0.01
	Magpie	0.3	0.034	0.016	0.2	0.003	0.001	0.037	0.02
	Osprey				0.7	0.01	0.005	0.010	0.00
Sarawak†	N/A	4	0.45	0.213				0.45	0.21
BDP total		7.18	1.52	0.71	23.5	0.35	0.16	1.9	0.88
						Average BDP rate since 15 Ma (Gt Myr^{-1})		0.12	0.06

*Total hydrocarbons in-place based on estimates of ultimate recoverable reserves and recovery factors for each field based on ref. 4.
†Estimated oil-in-place based on ref. 5.

Supplementary references

1. Green JAM, David TW. Non-assimilated tidal modeling of the South China Sea. *Deep Sea Res Part I* **78**, 42-48 (2013).

2. MacEachern JA, Bann KL. The role of ichnology in refining shallow marine facies models. In: *Recent Advances in Models of Siliciclastic Shallow-Marine Stratigraphy* (eds Hampson GJ, Steel RJ, Burgess PM, Dalrymple RW) (2008).

3. Ainsworth RB, Vakarelov BK, Nanson RA. Dynamic spatial and temporal prediction of changes in depositional processes on clastic shorelines: Toward improved subsurface uncertainty reduction and management. *AAPG Bull* **95**, 267-297 (2011).

4. Sandal ST. *The geology and hydrocarbon resources of Negara Brunei Darussalam*. Brunei Shell Petroleum Company, Brunei Museum (1996).

5. Abu Bakar M, *et al.* EOR Evaluation For Baram Delta Operations Fields, Malaysia. SPE Enhanced Oil Recovery Conference, Kuala Lumpur (2011).
